# Synchronous HPV-Related Cancer of Bilateral Tonsils Detected Using Transoral Endoscopic Examination with Narrow-Band Imaging

**DOI:** 10.1155/2017/9647010

**Published:** 2017-10-12

**Authors:** Fukuko Shimizu, Kenji Okami, Koji Ebisumoto, Daisuke Maki, Akihiro Sakai, Go Ogura, Naoya Nakamura, Masahiro Iida

**Affiliations:** ^1^Department of Otolaryngology-Head and Neck Surgery, Tokai University, Isehara, Japan; ^2^Department of Pathology, Tokai University, Isehara, Japan

## Abstract

**Background:**

The incidence of human papillomavirus- (HPV-) related oropharyngeal squamous cell carcinoma (OPSCC) has been rapidly increasing worldwide. HPV is reported in approximately 50% cases of OPSCC in Japan. However, there are few reports of synchronous bilateral HPV-positive tonsillar carcinoma, and, in almost all those cases, carcinoma was detected using positron emission tomography/computed tomography and/or bilateral tonsillectomy.

**Methods and Results:**

We report the case of a 63-year-old male with bilateral tonsillar carcinoma detected using transoral endoscopic examination with narrow-band imaging (NBI). A biopsy of the bilateral tonsils revealed squamous cell carcinoma, which was demonstrated to be HPV-related using in situ hybridization and p16 immunohistochemistry. The patient was diagnosed as synchronous bilateral tonsillar carcinoma: T1 (2) N2b M0. He was treated with induction chemotherapy, bilateral radical tonsillectomy with neck dissection, and radiotherapy.

**Conclusion:**

To our knowledge, this is the first report of a synchronous bilateral tonsillar carcinoma detected using transoral NBI in the outpatient setting. Early diagnosis without the inspection under general anesthesia is beneficial for the patients with lymph node metastasis from unknown primary lesion.

## 1. Introduction

Cervical metastases from an unknown primary site account for approximately 5%–10% of the head and neck cancers [1]. The standard protocol for investigating patients with unknown primary disease includes imaging with computed tomography (CT), magnetic resonance imaging (MRI), or both, followed by direct examination under general anesthesia and blind biopsy of potential primary sites, including nasopharynx, base of the tongue, hypopharynx, and ipsilateral tonsils. Fluorodeoxyglucose- (FDG-) positron emission tomography (PET)/CT is becoming an increasingly popular diagnostic tool; however, it has some limitations [2]. It is well known that the tonsils may be the site of an occult primary tumor. The incidence of occult tonsillar carcinomas has been reported to be 18%–39% [1, 3–6]. However, tonsillar carcinoma detection is likely to be missed if the lesion is small and submucosal or is present on the contralateral cervical node. Thus, several reports recommend bilateral tonsillectomy for the detection of the primary site [7].

There is approximately a 4% incidence of synchronous secondary tumors in the head and neck area [8]. A secondary tumor is characterized as synchronous if it is diagnosed concomitantly or within 6 months of the primary tumor [9]. Bilateral synchronous tonsillar carcinomas are rare, with only twenty case reports found in a literature review [10]. Almost all of those were detected using PET/CT or bilateral tonsillectomy.

Head and neck squamous cell carcinoma (HNSCC) is strongly associated with lifestyle, including smoking and alcohol consumption, and the overall incidence has been declining in the United States in the past 20 years [11]. This has been attributed to the decreasing prevalence of smoking [12, 13]. However, recently, human papillomavirus (HPV) infection has been identified as a new risk factor for a subset of oropharyngeal squamous cell carcinomas (OPSCCs), including those of the tonsils and base of the tongue [11–16]. During 2010–2016, eight cases of HPV-related synchronous bilateral tonsillar carcinoma have been reported [16–18]. The modalities used for carcinoma detection in these cases were PET/CT and/or bilateral tonsillectomy.

Recently, we recommended using narrow-band imaging (NBI) for the detection of primary lesions [19]. Transoral examination with NBI helps detect primary lesions in the tonsils, especially in HPV-related cases. This examination can be easily performed in the outpatient setting without any preparation [20]. Here, we report a rare case of synchronous HPV-related cancer of the bilateral tonsils. To the best of our knowledge, this is the first report of a synchronous bilateral tonsillar cancer detected using transoral NBI in the outpatient setting.

## 2. Case Report

**Figure 1:**
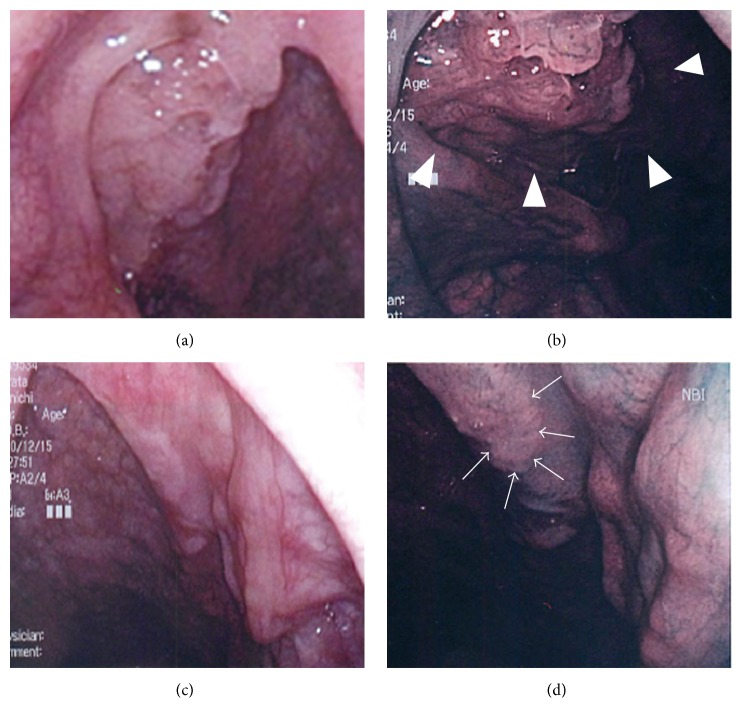
Endoscopic findings of palatine tonsils. The lesion could not be clearly visualized using conventional endoscopy ((a) right tonsil; (c) left tonsil). NBI enhanced an irregular surface and abnormal vascular proliferation ((b) right tonsil; (d) left tonsil).

**Figure 2:**
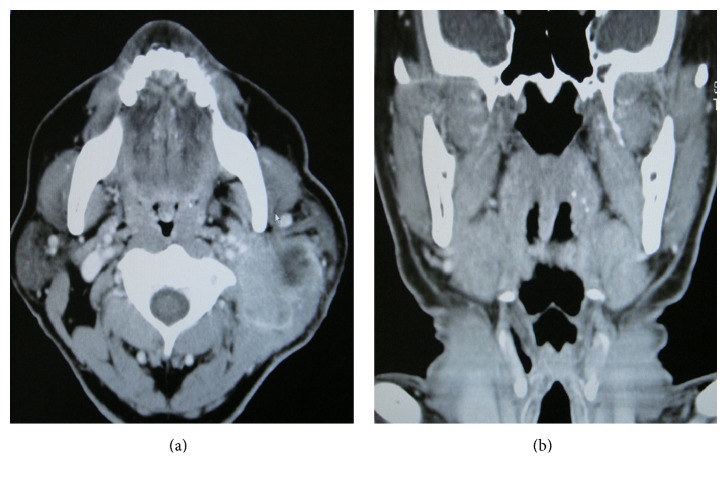
CT scan with contrast enhancement ((a) axial view; (b) coronal view). A CT scan demonstrated a cystic mass on the left side of the neck; however, tonsillar tumors were not detected.

**Figure 3:**
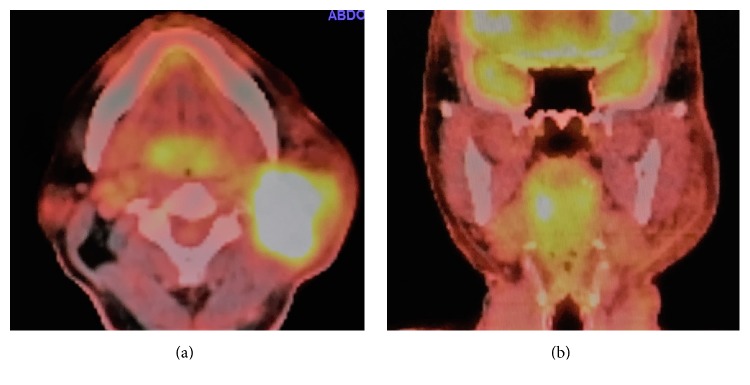
PET/CT scan ((a) axial view; (b) coronal view). A PET/CT showed a high accumulation of fluorodeoxyglucose (FDG) to the left neck mass, and there was a slight laterality of FDG accumulation in the tonsils with the right-side predominance. The tonsillar uptake of the FDG was within the physiological range, which was not conclusive for the diagnosis of the tonsillar primary lesion.

**Figure 4:**
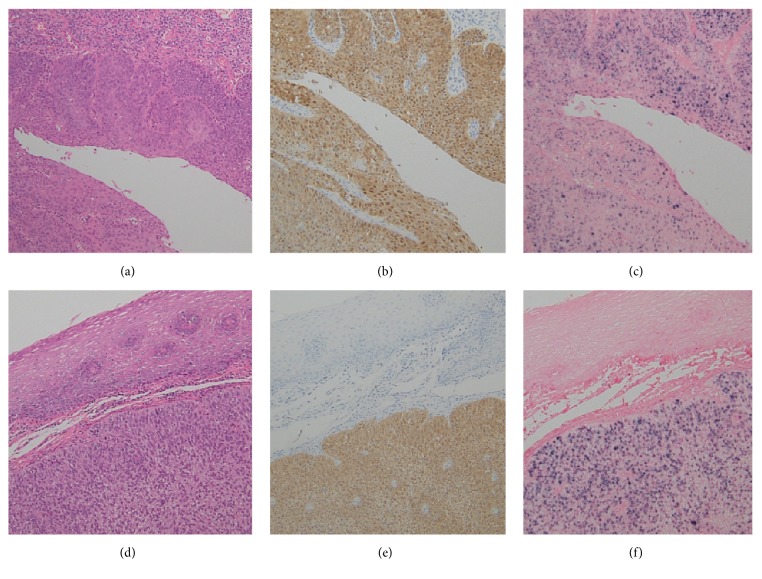
Histopathological results ((a), (b), and (c) right side; (d), (e), and (f) left side). Histopathological analysis of the biopsied tonsillar specimen revealed poorly differentiated SCC ((a) and (d)). Immunohistochemical staining for p16 was strongly positive in both lesions ((b) and (e)). ISH was positive for high-risk HPV ((c) and (f)).

A 63-year-old man presented with a 2-month history of a left neck mass. No obvious primary lesion was detected on routine otolaryngologic examination; however, fine-needle aspiration cytology revealed squamous cell carcinoma (SCC). He was referred to our hospital with an unknown primary neck metastasis. We sought the primary lesion throughout the pharynx; a small granular lesion on the right tonsil and a tiny mass on the left tonsillar pillar were recognized using transoral endoscopic examination with NBI (Figure 1). Both lesions could not be observed by transnasal endoscopic examination. CT revealed a lymph node swelling, 6 cm in size, invading the deep muscle on the left side of the neck. However, the tonsillar tumors were not detected (Figure 2). A PET/CT showed high accumulation of fluorodeoxyglucose (FDG) to the left neck mass (SUVmax = 12.0), and there was a slight laterality of FDG accumulation in the tonsils with the right-side predominance. However, the uptake of the FDG was within the physiological range (SUVmax = 5.6), which was not conclusive for the diagnosis of the tonsillar primary lesion (Figure 3). A biopsy of the bilateral tonsils revealed poorly differentiated SCC that was shown to be HPV-related using immunohistochemistry for p16 and in situ hybridization (ISH) (Figure 4). Polymerase chain reaction (PCR) was performed using primers targeting part of the L1 region of the HPV genome, revealing that all the samples contained HPV-16 DNA. Therefore, the patient was diagnosed with synchronous bilateral tonsillar cancer T1 (2) N2b M0 (UICC TNM Classification 7th edition). After three courses of induction chemotherapy (ICT) with Cisplatin (80 mg/m^2^, day 1) and 5-Fluorouracil (800 mg, days 1–5), the primary tumor showed a complete response (CR), and the neck lymph node showed a partial response (PR). The patient underwent bilateral radical tonsillectomy to confirm CR and underwent neck dissection. Histopathological examination revealed no primary tumor remnant in the bilateral tonsils and positive lymph node metastasis with extracapsular spread. Although we recommended the postoperative chemoradiotherapy covering the bilateral tonsillar beds and neck area, the patient refused the invasive treatment because of his job (professional cook). He was so concerned with the adverse events like xerostomia and taste change that we indicated the postoperative radiation therapy alone to the left neck (54 Gy). Approximately 15 months after the radiation therapy, he developed a regional relapse in the right lymph node. Thus, he underwent right neck dissection and has been alive without disease for 3 years after the salvage surgery.

## 3. Discussion

Joseph et al. reported that bilateral HPV-related tonsillar carcinoma is caused by the same HPV-16 variant [21]. In our reported case, the biopsy of the bilateral tonsils showed poorly differentiated SCC and ISH showed HPV positivity, indicating high risk in both the tonsils. PCR results showed the presence of HPV-16 DNA in both samples. This suggests that HPV infection type was the same in both tonsils. This could help clarify the underlying mechanisms of development of second primaries in HPV-related HNSCC.

Previous cervical cancer research has suggested three potential molecular mechanisms for the development of multifocal HPV-associated head and neck cancers [22].

HPV-related tonsillar cancers arise from the basal layer of the tonsillar crypt, and the tumor is often covered by normal mucosa, not exposed to the luminal surface at the early stage. Hence, the tumor growing inside the palatine tonsils may not be easy to visualize if the mucosal defect is too small. Therefore, transoral examination with NBI is helpful for detecting the primary lesion in the tonsils, especially in HPV-related cases. This examination can be performed easily in the outpatient setting without any complicated preparations [20].

In the case of lymph node metastasis from unknown primary lesion, bilateral tonsillectomy is recommended to detect the primary cancer in the tonsils. However, tonsillectomy needs general anesthesia, which may delay the diagnosis and treatment. In the present case, transoral NBI helped us diagnose the bilateral lesions at the outpatient clinic and initiate the treatment without delay.

Synchronous bilateral tonsillar carcinomas are very rare, with only 20 cases reported in the English literature prior to this case; furthermore, only eight cases of HPV-related synchronous bilateral tonsillar carcinoma have been reported since 2010 (Table 1). To the best of our knowledge, this is the first report of a synchronous bilateral tonsillar cancer detected using transoral NBI in an outpatient setting.

## 4. Conclusion

Transoral NBI examination is a useful method to detect the primary sites of unknown neck metastases. This is especially important in the era of increased incidence of HPV-related tonsillar cancers.

## Figures and Tables

**Table 1:** Previously reported cases of bilateral synchronous HPV-related tonsillar carcinoma since 2010.

Author	Year	Cases	Modality of investigation
Roeser	2010	1	Bilateral oropharyngectomy
McGovern	2010	3	Bilateral tonsillectomy
Joseph et al.	2013	1	Retrospective study
Nakahara et al.	2014	1	PET/CT
Rasband-Lindquist	2016	2	Unknown
